# Health Properties of Plant Bioactive Compounds: Immune, Antioxidant, and Metabolic Effects

**DOI:** 10.3390/ijms24097916

**Published:** 2023-04-26

**Authors:** Ivan Cruz-Chamorro, Antonio Carrillo-Vico

**Affiliations:** 1 Departamento de Bioquímica Médica y Biología Molecular e Inmunología, Facultad de Medicina, Universidad de Sevilla, 41009 Seville, Spain; 2 Instituto de Biomedicina de Sevilla, IBiS/Hospital Universitario Virgen del Rocío/CSIC/Universidad de Sevi-lla, 41013 Seville, Spain

In recent decades, people in the industrialized world have increased the demand for meat-free foods motivated by health, environmental, and animal welfare reasons. Thus, plant products and their derivatives have gained great popularity in nutrition [1]. Meanwhile, the World Health Organization (WHO) recommends the frequent consumption of plant foods instead of foods of animal origin, which contain a considerable amount of saturated fat and cholesterol [2].

The high consumption of plant products has generated an increase in the study of the beneficial properties of their components beyond their basic macro- and micro-nutrients [3,4]. In this context, several studies have shown that vegetable-derived peptides have multifunctional effects related to the main components of chronic diseases, which have attracted interest from the food, nutraceutical, and pharmaceutical industries.

In this Special Issue, several studies in which different vegetable extracts were tested have been compiled. The enzyme-assisted extraction of plant compounds is a widely used method and can be a strategy for sustainable and functional applications [5]. This method was used to produce an extract of *Castana sativa* (chestnut) that was shown to have antimicrobial activity against Streptococcus and Staphylococcus, among others [6]. These microorganisms are present in the oral cavity during oral mucositis, a common side effect of oncological treatment. Moreover, the high phenolic content of this extract has been shown to possess antioxidant activity. Other extracts of *Chenopodium quinoa* (quinoa), *Amaranthus retroflexus* (amaranth), and *Fagopyrum esculentum* (buckwheat), obtained by simulated gastrointestinal digestion, showed protective effects against IL-1-induced inflammation in vitro [7]. Additionally, an extract of *Protium heptaphyllum* gum resin has been shown to reduce cholesterol production in human liver cells and regulate the gene expression of several proteins involved in cholesterol metabolism [8].

Methyl p-coumarate, an esterified derivative of p-coumaric acid and a naturally occurring compound in plants, has been shown to be an effective agent for reducing inflammation in an experimental in vivo model of allergic asthma, reducing the influx of immune cells and mucus secretion in the lung, among other activities [9].

Another natural compound was also tested in a mouse model of anxiety. In that study, a protein hydrolysate from *Lupins angustifolius* (lupin) was able to reduce anxiety in mice evaluated by the elevated plus maze and Morris water maze behavior tests [10].

The clinical application of a vegetable extract of *Rosmarinus officinalis* (rosemary) was studied in four patients with osteogenesis imperfecta, a genetic connective tissue disease [11]. In this study, the extract reduced collagen accumulation in the fibroblasts of patients, due to increased autophagy of fibroblasts. Furthermore, the rosemary extract attenuated pro-apoptotic markers, such as cleaved caspase 3.

Finally, the biological effects of *Lycium barbarum* berries [12], the essential oils of *Annonaceae* species [13], antioxidant compounds involved in several disorders [14], as well as phytosterols in neurodegenerative diseases [15], and different natural compounds in the prevention and treatment of oral mucositis [16] were reviewed.

This Special Issue provides an overview of the current research being carried out with vegetable-derived bioactive compounds. All of these findings confirm and point out that these compounds may be possible new nutraceuticals capable of preventing or treating several diseases.
